# PTHrP Promotes RBP4 Expression Under the Control of PPARγ in the Kidney

**DOI:** 10.3390/ijms26010142

**Published:** 2024-12-27

**Authors:** María Paz Nieto-Bona, Almudena G. Carrasco, Gema Medina-Gomez, Ricardo J. Bosch, Adriana Izquierdo-Lahuerta

**Affiliations:** 1 Departamento de Ciencias Básicas de la Salud, Facultad de Ciencias de la Salud, Universidad Rey Juan Carlos, Avda. de Atenas s/n, 28922 Alcorcón, Madrid, Spain; paz.nieto@urjc.es (M.P.N.-B.); almudena.gcarrasco@urjc.es (A.G.C.); 2 Departamento de Biología de Sistemas, Facultad de Medicina y Ciencias de la Salud Nacional II, Universidad de Alcalá, Km. 33,600, 28805 Alcalá de Henares, Madrid, Spain; ricardoj.bosch@uah.es

**Keywords:** PTHrP, PPARγ, RBP4, kidney, podocyte

## Abstract

Parathyroid hormone-related protein (PTHrP) and retinol-binding protein 4 (RBP4) have been associated with a worse prognosis of kidney disease. Recently, the direct interconnection between PTHrP and the peroxisome proliferator-activated receptor gamma (PPARγ), a nuclear receptor whose activation is nephroprotective, has been discovered. The aim of this study was to analyze the relationship between PTHrP, PPARγ, and RBP4. For this purpose, we analyzed the levels of these proteins, which were studied in the kidneys of five experimental groups of mice at 6 weeks of age: controls, diabetics, insulin-treated diabetics, transgenic mice overexpressing PTHrP at the renal level, and the latter mice that were also induced with diabetes. In addition, we also analyzed the expression levels of these molecules in two mouse podocyte cell lines, controls and PPARγKO, subjected to a lipotoxic insult by palmitic acid. We found that RBP4 and PTHrP are increased in the kidney in pathological conditions and that insulin and PPARγ act regulating PTHrP and RBP4 expression, suggesting that the regulation of this system is critical for the maintenance of renal homeostasis and how it becomes imbalanced in different pathophysiological conditions.

## 1. Introduction

Retinol-binding protein 4 (RBP4) is a retinol (vitamin A) transporter protein in the human body. RBP4 expression is highest in the liver, but it is also found in adipose tissue and other organs, including the kidney [1,2,3]. This protein has been identified as a sensitive biomarker of renal tubular damage. Under normal conditions, RBP4 is filtered by the glomeruli and reabsorbed in the proximal tubules. However, in renal diseases, RBP4 levels in urine increase, indicating tubular damage. In addition, recent studies have shown that RBP4 may be involved in insulin resistance and diabetes, conditions that are often associated with diabetic nephropathy [3].

PPARγ is a nuclear receptor that regulates the expression of genes involved in lipid and glucose metabolism. In the kidney, PPARγ plays a protective role by reducing inflammation and fibrosis, processes that are critical in the progression of chronic kidney disease [2,4,5]. In recent years, it has been described that the retinol-binding proteins (RBP)1, RBP4, and RBP7 are target genes of PPARγ, showing the involvement of this nuclear receptor also in vitamin A metabolism [6,7,8,9,10].

Parathyroid hormone-related protein (PTHrP), on the other hand, is a protein which under physiological conditions is practically undetectable in blood but was discovered because of significant elevation in cancer in 1987. PTHrP is encoded by the *PTHLH* gene, located on autosome 12 in humans, and its transcription by alternative splicing produces three isoforms of 139, 141, and 173 residues, with distinct endocrine, paracrine, autocrine, and intracrine activities [11]. The N-terminal end of PTH and PTHrP are similar and allow binding through this domain to a common receptor, the PTH/PTHrP type 1 receptor (PTH1R) [12], to induce the cAMP/protein kinase A (PKA)/cAMP-response element-binding protein (CREB) pathway [11]. Intracrine actions of PTHrP have also been described, involving calcium signaling and promoting the activation of multiple genes via non-canonical pathways, as it occurs in cells that do not express PTH1R [13]. PTHrP is expressed in many tissues and its functions include regulating calcium transport in the kidney, bone, and placenta, smooth muscle tone, cell proliferation, cell differentiation, and cell death [12]. PTHrP overexpression is associated with worse prognosis in the disease, and in recent years, it has been shown to promote browning and lipolysis of adipose tissue [14,15]. In the kidney, PTHrP is expressed both at the glomerular and tubular levels and has been implicated in the regulation of renal function and in the pathogenesis. In this sense, PTHrP boosts tubulointerstitial cell survival by activating the PTH1R promoting anti-apoptotic factors via Runx2 [16]. It has also been shown that PTHrP contributes to the progression of renal damage, as it promotes macrophage infiltration, hypertrophy, epithelial–mesenchymal transition (EMT) [17], and fibrosis by activating TGF-β1 [11,18,19,20]. It has been shown to be up-regulated in several nephropathies [21,22,23], including diabetic nephropathy in humans and animal models [18,19,24,25].

Recently, it has been proposed that there is a reciprocal control relationship between PTHrP and PPARγ in adipose tissue [26]. Given the importance of both molecules in the correct functioning of the kidney and more specifically of the podocyte, the present article aims to delve into the possible relationship between both molecules at the renal level and proposes the involvement of retinol-binding protein 4 (RBP4), the target gene of PPARγ.

For this purpose, the expression of RBP4 has been studied in five experimental groups of mice: controls, diabetics, insulin-treated diabetics, mice that overexpress PTHrP at the renal level, and a group of diabetics. To complete the study and given the implication of these molecules in lipid metabolism, a lipotoxic insult, treatment with palmitic acid, has been tested on control and PPARγ-deficient mouse podocytes.

## 2. Results

### 2.1. PTHrP and Diabetes Promote an Increased RBP4 Expression in the Kidney

We wanted to know the renal expression of RBP4 at 6 weeks in five groups of mice. The data for these animals—body weight, kidney weight, glycemia, and proteinuria at the age of 6 weeks—are shown in Table 1.

**Figure 1:**
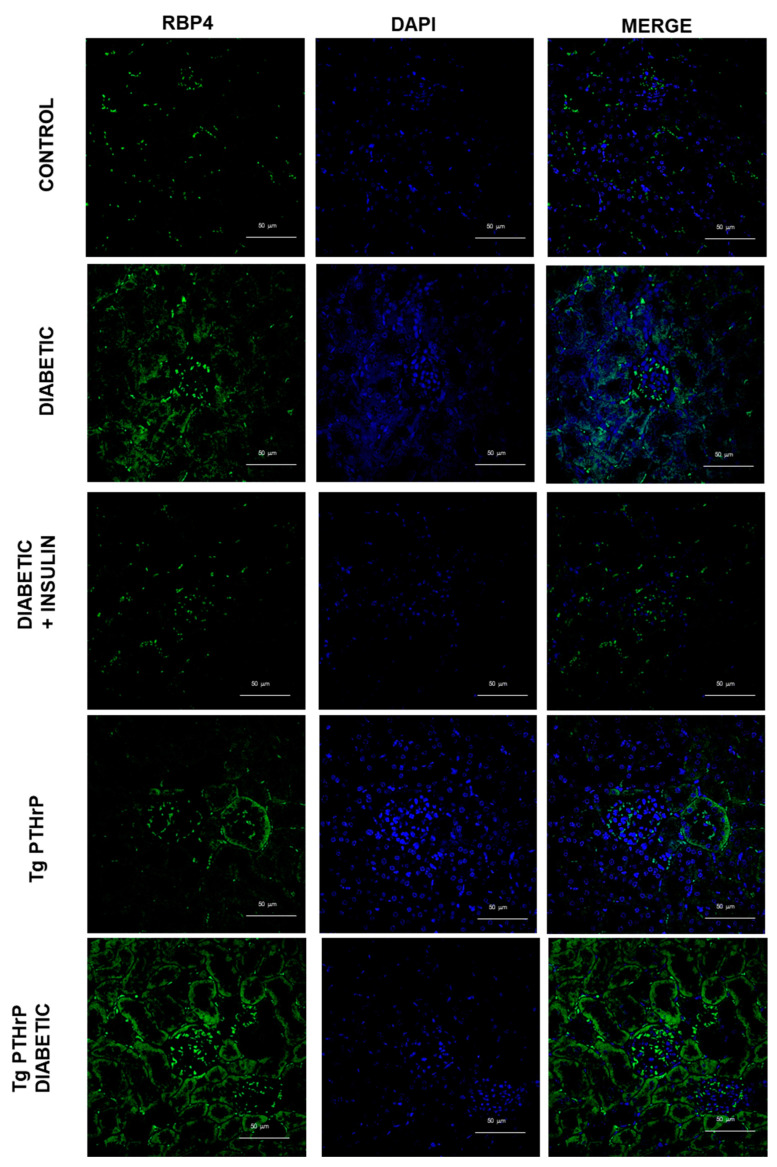
RBP4 expression is incremented in diabetic kidney and renal PTHrP overexpression promotes increased RBP4 expression. Representative micrographs of the immunofluorescence for RBP4 (green) in kidney of mice: control, diabetic, diabetic treated with insulin, Tg PTHrP, and Tg PTHrP diabetic. PTHrP: parathyroid hormone-related protein; RBP4: retinol-binding protein 4; Tg PTHrP: transgenic overexpression of PTHrP. Magnification 400×.

**Figure 2:**
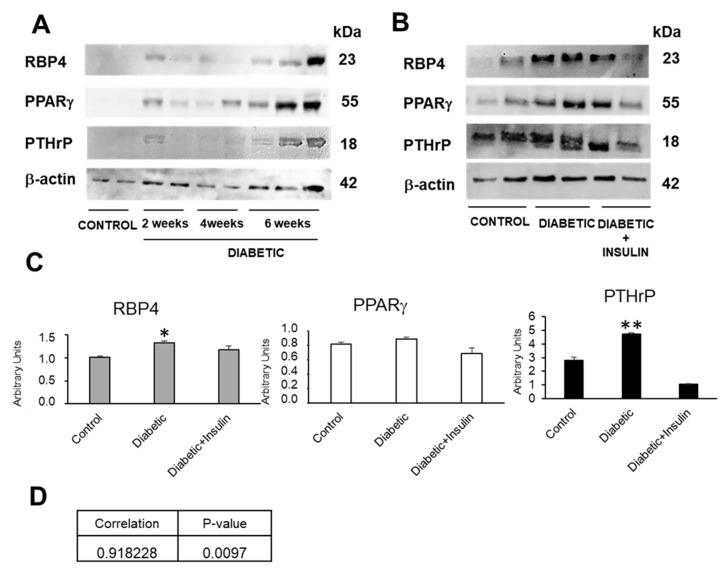
RBP4 and PTHrP expression is incremented in diabetic kidney and insulin treatment prevent this increase, while PPARγ is not changed at this age. (**A**) Representative western blot of RBP4, PTHrP, and PPARγ in kidney of diabetic mice of 2, 4, and 6 weeks of age; (**B**) representative western blot of RBP4, PTHrP, and PPARγ in kidney of control mice, diabetic mice, and diabetic mice treated with insulin at 6 weeks. (**C**) Quantifications of protein expression in kidney of mice of different groups at 6 weeks. (**D**) Pearson correlation between PTHrP and RBP4 protein levels expression in mice. N = 6–8 animals/group; PPARγ: peroxisome proliferator-activated receptor gamma; PTHrP: parathyroid hormone-related protein; RBP4: retinol-binding protein 4; * *p* < 0.05; ** *p* < 0.01.

For this purpose, we analyzed the renal expression of RBP4 by immunofluorescence (Figure 1) and western blot (Figure 2).

Compared with the controls, diabetic animals show increased RBP4 renal expression as the disease progresses, presenting a maximum expression at 6 weeks. This experimental time was chosen to continue the research (Figure 2A). However, this RBP4 renal expression decreases after treatment with insulin, at 6 weeks of age (Figure 2B). Our laboratory had previously shown that diabetic animals [25,27] showed an increase in PTHrP expression associated to proteinuria at the renal level. In addition, we also found that insulin treatment prevented this PTHrP increase (Figure 2B).

When we analyze the expression of RBP4 in animals overexpressing PTHrP in the kidney, we find a notable increase compared to control animals, which becomes more evident when these animals are also diabetic (Figure 1).

Since RBP4 is a target gene of PPARγ, we wanted to know the renal expression of this nuclear receptor in these diabetic animals and the possible therapeutic action of insulin treatment.

At 6 weeks of age, the diabetic animals showed no significant changes in PPARγ that were at least partially recovered in those treated with insulin (Figure 2A,B). In the case of PTHrP the results were the opposite, since it increased significantly in the 6-week-old diabetic animals and exhibited partially recovered values close to the controls in the diabetic animals injected daily with insulin (Figure 2A,B).

**Figure 3:**
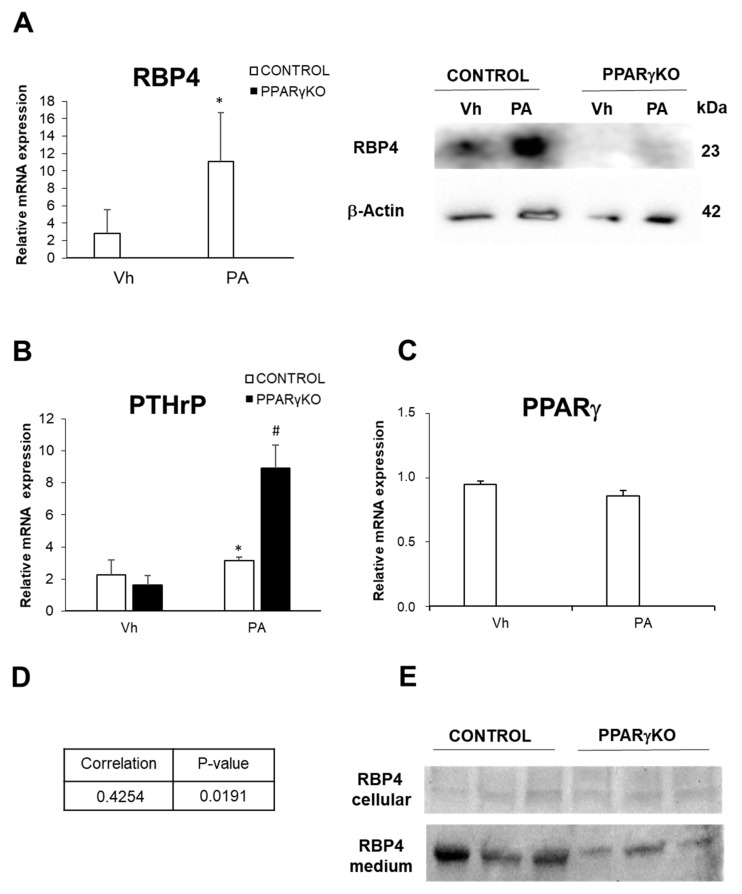
Expression of RBP4, PTHrP, and PPARγ in control and PPARγKO podocytes. (**A**) Relative mRNA expression of and protein expression of RBP4; (**B**) relative mRNA expression of PTHrP; (**C**) relative mRNA expression of PPARγ; (**D**) Pearson correlation between PTHrP and RBP4 mRNA levels expression in control podocytes; (**E**) RBP4 detection in supernatant of control and PPARγKO podocytes cultures. Data are shown as mean ± SEM (n = 3 experiments). * *p <* 0.05 versus Vh control podocytes; # *p <* 0.05 versus Vh PPARγKO podocytes. Vh: vehicle; PA: palmitic acid. N = 6–8 animals/group; PPARγ: peroxisome proliferator-activated receptor gamma; PTHrP: parathyroid hormone-related protein; RBP4: retinol-binding protein 4.

Since it has been described in the lungs that PPARγ can bind to the PTHrP gene [28] as a repressor and that, in turn, PTHrP is able to decrease the ability of PPARγ to activate its target genes, without affecting their levels of expression [29], we wanted to check if there was a possible correlation between the protein levels of both factors at the renal level, but we found no correlation between PPARγ and PTHrP. However, we did find a positive correlation between renal protein values of RBP4 and PTHrP (Figure 3).

### 2.2. PA Stimulates RBP4 and PTHrP Expression in Podocytes

In addition, and given the observed increase in RBP4 in animal diabetic models and the additive effect of PTHrP overexpression, we wanted to know if other damages exerted on podocytes, key cells of the glomerular filtration barrier, such as the addition of palmitic acid, would affect the expression of RBP4 in these and would also correlate with the expression of PTHrP and PPARγ. For this purpose, we used two mouse podocyte lines, control and PPARγ-defective (PPARγKO) podocytes. Our group has previously shown that RBP4 expression was greatly decreased in PPARγKO podocytes [2].

We found that control podocytes treated with PA showed an increased expression of RBP4 (Figure 3A) and PTHrP (Figure 3B), while PPARγexpression showed no significant changes (Figure 3C).

Furthermore, when we analyzed mRNA levels expression of PTHrP and RBP4, we found its correlate in control podocytes (Figure 3D).

### 2.3. PPARγ Deficiency in Podocytes Promotes a Significant Increase in PTHrP and a Decrease in RBP4 Expression Following Lipotoxic Damage by Palmitic Acid

When PPARγKO podocytes were subjected to PA lipotoxic insult, the change was that PTHrP increased three-fold. When we analyzed the podocyte culture media, we found that both cell types excreted RBP4 and that in the case of control podocytes this excretion was notably higher than in PPARγKO podocytes (Figure 3A).

Despite the remarkable increase in PTHrP in this cell type, these PPARγ-deficient cells showed a very low expression of RBP4 both in normal conditions (data already published by our laboratory) and after treatment with palmitic acid (Figure 3A).

### 2.4. Podocytes Excrete RBP4

RBP4 is a protein that is secreted to the outside of the cell for the transport of retinol. We wanted to know if podocytes were able to excrete this protein and for this purpose, we analyzed the supernatant of podocyte cultures. In Figure 3E, we can see that both control and PPARγKO podocytes excrete RBP4 into the medium. However, as mentioned above, RBP4 expression is much lower in these PPARγKO podocytes (Figure 3E).

## 3. Discussion

The interactions between RBP4, PPARγ, and PTHrP in the kidney are complex and multifaceted. Our results show that RBP4 and PTHrP levels are elevated in both diabetes and lipotoxic PA damage in podocytes. Our results also show that PTHrP overexpression promotes RBP4 elevation in the kidney and how these two factors correlate in the whole kidney and in podocytes, probably contributing to renal damage in pathological conditions. In this sense, it has also been described in chondrocytes that the addition of PTHrP promotes the excretion of RBP4 to the medium [30].

Since it has been described that PTHrP overexpression is related to a worse prognosis of renal disease [15,25,31]. Specifically, studies have shown that overexpression of PTHrP in the kidney is associated with cancer, but also renal hypertrophy and proteinuria, two hallmarks of diabetic nephropathy. In addition, PTHrP can modulate glomerular hemodynamics and participate in renal inflammation and fibrosis, exacerbating renal damage under conditions of hyperglycemia [19,22,24,25].

It has also been described that increased levels of RBP4 are related to pathological conditions (diabetes, obesity, heart disease) [1,3]. Concretely, elevated levels of RBP4 in urine have been associated with various renal pathologies, including glomerulopathies, diabetes, renal allograft dysfunction, chronic kidney disease, and preeclampsia. However, the expression of RBP4 is also modulated by the nuclear receptor PPARγ, which when activated acts as a nephroprotector [2,4], but, in our study, PPARγ levels are not elevated and tend to decrease without being significant in the diabetic kidney and in lipotoxic PA damage. This would explain the lack of protective effect of PPARγ against the “triggering” of PTHrP that contributes to the damage and that in turn seems to promote the increase in RBP4, a deleterious molecule. Furthermore, these increased PTHrP levels due to the lack of activation of PPARγ is supported by the fact that its deficit in PPARγKO podocytes triggers the expression of PTHrP, but not of RBP4, a target gene of PPARγ which requires the presence of this nuclear receptor for its own expression.

Furthermore, in adipose tissue it has been proposed that there is an opposite regulation between PTHrP and PPARγ [26], since it has been shown that both factors are opposite in the regulation of mesenchymal stem cells towards adipogenesis or osteogenesis [29,32,33]. Furthermore, in the lungs it has been shown that PPARγ is able to bind to the gene coding for PTHrP (*Pthlh*) and its expression significantly increased embryonic development in specific *Ppar*γ conditional knockout mice [28]. On the other hand, PTHrP can inhibit the ability of PPARγ to exert its effect on target genes, without affecting their transcription levels [29].

**Figure 4:**
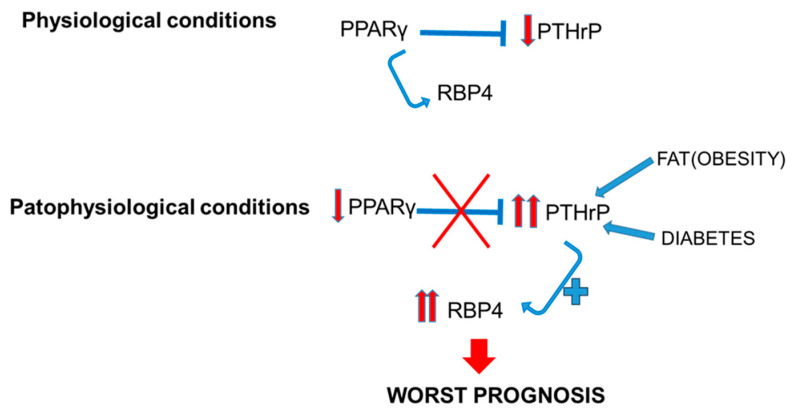
Relation between PTHrP and PPARγ in the kidney, and implications of RBP4 in physiological and pathophysiological conditions. PPARγ: peroxisome proliferator-activated receptor gamma; PTHrP: parathyroid hormone-related protein; RBP4: retinol-binding protein 4.

All these effects are what we believe to be occurring in the kidney as well (Figure 4). Under physiological conditions, PTHrP levels in the kidney remain stable, as is the case for PPARγ and RBP4. However, in renal disease conditions such as diabetic nephropathy, obesity, or cancer, PTHrP increases markedly [14,25,31,34], as does RBP4 [1,3]. These effects could be explained by the lack of PPARγ repression on the PTHrP gene, since in all these diseases it is known that the levels of this nuclear receptor are decreased. This lack of repression of PTHrP would favor its synthesis, which in turn would promote the elevation of RBP4 levels, a serum protein, which would favor the promotion of damage (Figure 4).

In conclusion, there appears to be a direct relationship between PTHrP and RBP4 levels in the kidney, which increase in pathological conditions, and both molecules are in turn regulated by the action of PPARγ. This suggests that the regulation of this system is critical for the maintenance of renal homeostasis and how it becomes imbalanced in different pathophysiological conditions.

However, understanding these interactions is crucial for the development of new therapies aimed at mitigating kidney damage and improving renal function in patients with kidney disease. Thus, it is also necessary to further investigate the mechanisms and pathways involved in the relationship between these three factors, PTHrP, PPARγ, and RBP4, because although our laboratory has shown that the PTH1R receptor mediates the effects of PTHrP in diabetic nephropathy, the connection with RBP4 and PPARγ should be explored. Future studies are necessary to clarify this mechanism, since it is very likely that there are many other factors (receptors, second messengers, co-repressors, etc.) that are contributing to the final picture that we observe in renal disease.

## 4. Materials and Methods

### 4.1. Animals

Animals were housed at a density of four animals per cage in a temperature-controlled room (20–22 °C) with 12 h light–dark cycles. Male mice of five experimental genotypes were placed at weaning (3 weeks of age) on a normal chow diet. Food and water were available ad libitum unless noted.

Diabetes was induced by three consecutive daily intraperitoneal injections of STZ (Sigma, St. Louis, MO, USA), 65 mg/kg body weight in citrate buffer, with pH 4.5 (vehicle). A group of diabetic mice received daily subcutaneous injections of slow insulin (100 I.U./mL) (Lilly, Madrid, Spain) to maintain normal blood glucose concentrations until the day of sacrifice.

PTHrP-overexpressing mice were generously supplied by Professor A.F. Stewart and Dr. A. García-Ocaña (University of Pittsburgh School of Medicine, Pittsburgh, PA, USA) [22,25]. The renal specificity of the transgene was conferred by the g-glutamyl transpeptidase-l promoter, mainly expressed in the renal proximal tubule. PTHrP-overexpressing mice were generated by breeding two types of transgenic mice: one containing a g-glutamyl transpeptidase-l promoter fragment upstream of a tetracycline transactivator fusion protein, which functions as a strong transcription activator; and the other with a PTHrP complementary DNA placed under the control of a tetracycline operator construct. Transgene-bearing founders were continuously outbred to normal CD-1 mice to generate hemizygotes. Genotyping of these mice was performed by PCR, as described in [25].

Animals were individually housed in metabolic cages with free access to tap water, and 24 h urine was collected for protein. After the experimental period (2, 4, or 6 weeks), the mice were killed. Blood was taken by cardiac puncture and plasma glucose was determined by the glucose oxidase. The right kidney was removed, weighed, and frozen in liquid nitrogen for subsequent total RNA and protein extraction; the left kidney was fixed in 4% buffered p-formaldehyde for morphological and immunochemistry studies. All animal protocols used in this study were approved by the Universidad Rey Juan Carlos Research Ethics Committee (Ref. No. 1705202323323).

### 4.2. Cell Lines, Culture, and Treatments

We used a conditionally immortalized mouse PPARγ-floxed podocyte cell line and PPARγKO podocyte obtained by infection of the Cre recombinase adenovirus vector, which were obtained in the laboratory of Professor Richard Coward, as described by Carrasco et al. [2]. Both podocyte lines proliferate at 33 °C and become quiescent and differentiate when thermo-shifted to 37 °C. Differentiation requires 10–14 days.

All experiments were performed after 12 h of serum starvation. Passage numbers 6 to 12 were used for all experiments and were performed at least 3 times.

Palmitate (PA) treatment was performed as described previously [35]. Briefly, 20% fatty acid-free BSA solution was heated to 37 °C before the addition of a 100 mM PA stock solution dissolved in ethanol. The solution was heated to 37 °C until clear and diluted with RPMI 1640 to obtain a final concentration of 5% BSA, 1% ethanol, and 500 μM PA. The solution was filter-sterilized (0.2 µm pore size) before being added onto the cells. The control for palmitate (vehicle) was RPMI 1640, 5% fatty acid-free BSA, and 1% ethanol.

### 4.3. mRNA Extraction and Quantitative RT-PCR

RNA extraction, quantification, and retro-transcription was performed as described [21]. cDNA was also prepared from the conditionally immortalized mouse podocyte cell line, used for in vitro experiments. All quantitative RT-PCR assays were performed in duplicate for each sample. β-actin, 36b4, and 18s were used as housekeeping genes. To validate housekeeping genes, we used the BestKeeper software tool (version 1) [23]. All primers are listed in Table S1.

### 4.4. Protein Extraction and Western Blotting

Kidneys were homogenized in RIPA buffer, supplemented with a protease inhibitor cocktail (Sigma-Aldrich). Culture cells were washed twice with ice-cold PBS and scraped into RIPA. Protein concentration was determined by the Bradford method. Proteins were separated on 10 or 12% SDS-PAGE and transferred to nitrocellulose membranes. Membranes were blocked with 5% BSA (Sigma) in TBS-Tween 20 (TBS-T) followed by incubation with one of the following primary antibodies: rabbit polyclonal anti-PTHrP antiserum C6, recognizing the highly conserved C-terminal epitope 107–111 in the intact PTHrP molecule [18] at 1:2000 dilution, and anti-PPARγ (Santa Cruz Biotechnology Inc., Dallas, TX, USA) at 1:1000 dilution, anti-RBP4 (at 1:1000 dilution), and β-actin (Abcam Inc., Waltham, MA, USA) at 1:5000 dilution, overnight at 4 °C. After three washes in TBS-T, membranes were probed with the appropriate secondary antibody conjugated to the horseradish peroxidase (HRP), anti-mouse IgG (H + L)-HRP or anti-rabbit IgG (H + L)-HRP (Bio-Rad Laboratories, Inc., Hercules, CA, USA), both at 1:5000 dilution. The blots were visualized by using a chemifluorescent detection system (ClarityTM ECL Substrate, Bio-Rad Laboratories, Inc.) and the ChemiDocTM System (BioRad Laboratories, Inc.). The protein band density was measured using the ImageJ 1.45 software (National Institutes of Health, Bethesda, MD, USA). The amount of protein under control conditions was assigned a relative value of 100%.

### 4.5. Statistical Analysis

All data are expressed as mean values ± standard mean error (SEM) and n represents the number of animals, or cells experiments. Statistical significance was assessed by the Kruskal–Wallis test or Mann–Whitney test, when appropriate. Correlations were evaluated by Pearson’s r. The GraphPad InStat software 3.0x (GraphPad Software, Inc., Boston, MA, USA) was used. A *p* < 0.05 was considered significant.

## 5. Conclusions

There is a direct relationship between PTHrP and RBP4 levels in the kidney, which increase in pathological conditions, and both molecules are in turn regulated by the action of PPARγ. However, understanding these interactions is crucial for the development of new therapies aimed at mitigating kidney damage and improving renal function in patients with kidney disease.

## Figures and Tables

**Table 1:** Characteristics of the five experimental groups of mice at 6 weeks of age. Data are means ± s.e.m.; n = 6–8 in each group; * *p* < 0.05; ** *p* < 0.01.

Experimental Group	Body Weight (g)	Kidney Weight (g)	Glucose (mg/dL)	Proteinuria (mg/24 h)
Control	33.9 ± 0.1	0.23 ± 0.00	119.2 ± 4.7	1.9 ± 0.4
Diabetic	31.7 ± 0.2	0.30 ± 0.03 *	606 ± 60 **	10.5 ± 1.2 *
Diabetic with insulin	34.5 ± 1.0	0.29 ± 0.01	232 ± 17.1 *	9.0 ± 3.0
Tg PTHrP	32.8 ± 2.2	0.21 ± 0.01	133.3 ± 13.5	2.2 ± 0.4
Tg PTHrP diabetic	28 ± 1.1	0.33 ± 0.01 *	620 ± 58 **	12.3 ± 1.2 *

## Data Availability

Data is contained within the article and Supplementary Materials.

## Supplementary Materials

The supporting information can be downloaded at https://www.mdpi.com/article/10.3390/ijms26010142/s1.
