# Supplementary material for: PTHrP Promotes RBP4 Expression Under the Control of PPARγ in the Kidney
Source: Int J Mol Sci. 2024 Dec 27;26(1):142. doi: 10.3390/ijms26010142 (PMC11719952; doi:10.3390/ijms26010142)
Supplement: Supplementary file 1 [file ijms-26-00142-s001.zip › ijms-3377451-supplementary.pdf]

Table S1. Primer's sequence used in RT-PCR.

Gene	Forward	Reverse
Pthrp	TACAAAGAACAGCCACTC	GATCCCAATGCATTTACAGT
Pparg	TTTAAAAACAAGACTACCCTTTACTGAAATT	AGAGGTCCACAGAGCTGATTCC
Rbp4	GCTTCTACTGAGGAGCTGTTGTG	GGACGATCAACCACTCGGCCATTG
